# Half a World Apart? Overlap in Nonbreeding Distributions of Atlantic and Indian Ocean Thin-Billed Prions

**DOI:** 10.1371/journal.pone.0125007

**Published:** 2015-05-27

**Authors:** Petra Quillfeldt, Yves Cherel, Juan F. Masello, Karine Delord, Rona A. R. McGill, Robert W. Furness, Yoshan Moodley, Henri Weimerskirch

**Affiliations:** 1 Justus Liebig University Giessen, Department of Animal Ecology and Systematics, 35392, Giessen, Germany; 2 Centre d’Etudes Biologiques de Chizé, UMR 7372 CNRS-Université de La Rochelle, 79360, Villiers-en-Bois, France; 3 Life Sciences Mass Spectrometry Facility, Scottish Universities Environmental Research Centre, East Kilbride, Glasgow, G75 0QF, United Kingdom; 4 College of Medical, Veterinary and Life Sciences, University of Glasgow, Glasgow, G12 8QQ, United Kingdom; 5 Konrad Lorenz Institute for EthologyUniversity of Veterinary Medicine Vienna, A-1160, Wien, Austria; Nagoya University, JAPAN

## Abstract

Distant populations of animals may share their non-breeding grounds or migrate to distinct areas, and this may have important consequences for population differentiation and dynamics. Small burrow-nesting seabirds provide a suitable case study, as they are often restricted to safe breeding sites on islands, resulting in a patchy breeding distribution. For example, Thin-billed prions *Pachyptila belcheri* have two major breeding colonies more than 8,000 km apart, on the Falkland Islands in the south-western Atlantic and in the Kerguelen Archipelago in the Indian Ocean. We used geolocators and stable isotopes to compare at-sea movements and trophic levels of these two populations during their non-breeding season, and applied ecological niche models to compare environmental conditions in the habitat. Over three winters, birds breeding in the Atlantic showed a high consistency in their migration routes. Most individuals migrated more than 3000 km eastwards, while very few remained over the Patagonian Shelf. In contrast, all Indian Ocean birds migrated westwards, resulting in an overlapping nonbreeding area in the eastern Atlantic sector of the Southern Ocean. Geolocators and isotopic signature of feathers indicated that prions from the Falklands moulted at slightly higher latitudes than those from Kerguelen Islands. All birds fed on low trophic level prey, most probably crustaceans. The phenology differed notably between the two populations. Falkland birds returned to the Patagonian Shelf after 2-3 months, while Kerguelen birds remained in the nonbreeding area for seven months, before returning to nesting grounds highly synchronously and at high speed. Habitat models identified sea surface temperature and chlorophyll a concentration as important environmental parameters. In summary, we show that even though the two very distant populations migrate to roughly the same area to moult, they have distinct wintering strategies: They had significantly different realized niches and timing which may contribute to spatial niche partitioning.

## Introduction

Migratory species undertake regular seasonal movements to and from nonbreeding sites, thus spending parts of the year in widely separated and ecologically disparate environments. Migratory behaviour is especially widespread in birds (e.g. [1]), which show a high variability in migratory connectivity. In species with high migratory connectivity, individuals from different breeding areas mix during the nonbreeding season and vice versa. In contrast, other species have populations using well-defined, non-overlapping breeding and non-breeding areas, thus showing low migratory connectivity (reviewed by [2]). The degree of migratory connectivity may explain carry over effects from one season to the next, as well as differences in ability of to respond to selective pressures [2], and thus have implications for the ecology, evolution and conservation of migratory species (e.g. [3]).

Migration in marine birds, it is often characterised by long distance movements [4–7]. For seabirds, oceans provide large feeding habitats, interspersed with relatively few breeding sites on islands, resulting in a patchy breeding distribution. Procellariiformes (petrels, shearwaters and albatrosses) are the most pelagic of the seabirds, and nearly all species are migratory to some degree [8]. However, definitive information on their migration routes, travel distances, staging and nonbreeding areas are known for only a small minority (e.g. [9]). Migration routes of albatrosses and large petrels cover huge distances (*e*.*g*. 64,000 km in sooty shearwaters *Puffinus griseus* Gmelin, 1789; [4]). The few species that have been studied in detail show conserved temporal and general movement patterns, combined with considerable variability in the use of specific wintering areas within and among individuals. For example, sooty shearwaters breeding in New Zealand travelled across the equator to the North Pacific [4]. Similarly, sooty shearwaters from the Falkland Islands migrated to the North Atlantic [10]. The timing of the movement trajectory between the southern hemisphere breeding sites and northern hemisphere wintering areas was very similar in birds from Pacific and Atlantic breeding colonies yet the two populations displayed contrasting patterns of migratory connectivity. Birds from the Falklands showed high migratory connectivity: they settled in a single area in the northwest Atlantic for most of the austral winter [10]). In contrast, sooty shearwaters in the Pacific showed low migratory connectivity: birds from two breeding colonies mixed and used three discrete nonbreeding areas (off Japan, Alaska and California, [4]).

Thus, while populations of some procellariiform species may migrate to discrete non-breeding areas, populations of other species may share non-breeding areas completely or partially among conspecifics. It has been suggested that migration behaviour can profoundly influence population genetic structure: almost all seabird species with two or more population-specific non-breeding areas were phylogeographically structured [11], while other species were not. Thus, migration behaviour may be an important determinant of the taxonomic and conservation status of seabird populations, as threats in the breeding and wintering ranges as well as along the migratory routes may determine population persistence.

Miniaturized geolocators or Global Location Sensor (GLS) loggers now enable researchers to follow the at-sea movements of medium to small petrels (e.g. [12–14]). Thin-billed prions *Pachyptila belcheri* (Mathews, 1912) have two major breeding colonies, over 8,000 km apart in the Atlantic and in the Indian Ocean. In a previous study that tracked the non-breeding movements of thin-billed prions breeding on the Falkland Islands (Atlantic) nineteen out of 20 birds migrated to an area >3,000 km east of their breeding site [15]. However it was not known whether this population consistently used the same non-breeding areas from one year to the next. Furthermore, the migration routes of thin-billed prions from other colonies, including the major breeding site at Kerguelen in the Indian Ocean, were unknown. Using stable isotopes from adult feathers, Cherel *et al*. [16, 17] inferred a moulting area in Antarctic waters from consistently highly negative carbon stable isotope ratios of Kerguelen birds, which are very similar to the values observed among Falklands birds [18]. As the Falkland birds migrated eastwards to the Indian Ocean sector, this may indicate a common overwintering area, or, alternatively, that thin-billed prions breeding on Kerguelen spend their winters at similar latitudes, but in different areas.

To elucidate these patterns, which could be of genetic and conservation relevance for both populations, we conducted (1) an inter-year comparison of Thin-billed prions from the Falkland Islands over three winters and (2) an inter-population comparison of migratory movements of thin-billed prions from each of the two main breeding sites. Using geolocation loggers, we specifically aimed to: (i) compare migration routes and the timing of migratory movements of birds breeding in the Atlantic and in the Indian Ocean, (ii) detect any possible overlap in the wintering areas used by the two main colonies of the species, and (iii) examine annual variation in timing and destinations of migratory movements. The isotopic niche of the two populations during moult was investigated to provide complementary information on diet during the non-breeding period. Feathers reflect the diet at the time they were grown, because keratin is inert after synthesis [19, 20]. In thin-billed prions, adults do not moult while attending the breeding site and birds return to the colony in spring with moult completed. The adult moult is presumed to occur after completion of the breeding cycle, mainly from March to May [21]; hence feather stable isotope values are likely to reflect the autumn foraging ecology of the species.

## Materials and Methods

### Study species and sites

Thin-billed prions breed on islands off South America and in the Indian Ocean; there are several million birds in the Falkland and Kerguelen islands, a smaller population on Isla Noir (southern Chile) and a very small number (10–20 pairs) on the Crozet Islands [21]. They show the typical procellariiform pattern of a single-egg clutch and slow chick development. Thin-billed prions feed mainly on crustaceans during the breeding season and show some flexibility in diet within and between years [16, 17, 22].

To investigate spatial movements, we attached small leg-mounted geolocators (MK10, developed by British Antarctic Survey, Cambridge, UK) to breeding adult thin-billed prions over three years at New Island, Falkland/Malvinas Islands (51°43′S, 61°18′W) and one year at Île Mayes, Kerguelen (49°28’S, 69°57’E, for sample sizes, see Table 1). All animal work has been conducted according to relevant national and international guidelines. All sampling procedures and manipulations were reviewed or specifically approved as part of obtaining the field permit. Access to private land, field procedures and animal manipulations were approved by the New Island Conservation Trust, the Falkland Islands Government (Environmental Planning Office), the Animal Ethic Committee of the Institut Polaire Français Paul Emile Victor), and by the Préfet des Terres Australes et Antarctiques Françaises.

**Table 1:** Geolocator deployment and recovery times and sample sizes for thin-billed prions from the Falkland Islands (FLK) in 3 years and Kerguelen (KER) in 2012.

Year (Island)	Deployment (N, dates)		Recovery(N, dates)		Year-round tracks
2010 (FLK)	25	27/11/09–11/2/2010	20	17/12–29/12/2010	20
2011 (FLK)	20	25/12–31/12/2010	14	04/12–11/12/2011	9
2012 (KER)	29	13/01–18/01/2012	19	26/11–03/12/2012	15
2013 (FLK)	20	10/12–19/12/2012	11	29/11–14/12/2013	6

Nests were selected according to accessibility, and at New Island, the presence of individuals known from previous years, to maximize the chances of recapture. The birds were captured by hand at marked nests during incubation. The geolocators weighed 1 g (<1% of the mean body mass—130 g—of thin-billed prions) and were fixed to plastic leg bands. Tagged individuals were marked with numbered steel rings on the other leg. A blood sample for sex determination was taken from the wing vein and stored on Whatman FTA Classic cards. Burrows were revisited and devices retrieved during incubation in the following season (Table 1).

In the present analysis, we included data from a single winter period for each individual. Because several loggers stopped recording several months before device recovery, the final samples sizes for year-round tracks were smaller than for recovered data sets. Moreover, some return trips could not be determined as they were influenced by equinox uncertainties. As described previously [15], the return of Falkland birds to the breeding area was variable, taking 5 to 177 days (median = 13 days), because while most birds returned between April and June, others visited an intermediate nonbreeding area for variable time spans (range = 50–145 days), and a small number remained until early September. Thus, for the comparison with return flights of Kerguelen birds, we included all birds with a clearly directed, approximately linear return migration trip.

A detailed study found no evidence for any substantial impact of the geolocators on thin-billed prions: breeding performance was unaffected in the season of attachment or following recovery; eco-physiological measurements suggested that adults adapted to the higher load; and the similarity in stable isotope ratios in blood and feathers of instrumented adults and controls indicated that general diet and distribution was unaffected [23].

### Data processing

Geolocators provide two positions per day based on light levels, with an accuracy of approximately 186 ± 114 km [24]. Light data were analysed using the BASTrak software suite (British Antarctic Survey, Cambridge, UK). TransEdit was used to check for integrity of light curves and to determine dawn and dusk times, and Locator to estimate latitude from day length and longitude from the time of local mid-day relative to Greenwich Mean Time. We assumed a sun elevation angle of -3.5°, based on known positions obtained during pre- and post-deployment calibration of the loggers at the colony. All estimated locations were examined visually in a geographical information system (GIS) and any unrealistic positions—either associated with interference to light curves at dawn or dusk, or in temporal proximity to equinoxes when latitudes are unreliable—were excluded from further analyses. This leads to a reduced number of observations for the periods around the equinoxes. We also kept the unfiltered data, and these were used to obtain information about longitudinal movements during the equinox times, e.g. to define the timing of return migration that partly overlapped with equinox times. The timing of migration was determined from directed longitudinal movements that finished at or beyond the breeding colony longitude (S1 Fig). Outward migration timing was clearly distinguished in all migrating individuals, while the return migration was only determined in those individuals exhibiting a typical, clearly distinguishable migration pattern (2010:16 out of 19, 2011: 9 out of 9, 2012: 12 out of 15, 2013: 5 out of 6).

Centroid positions of the distribution in the nonbreeding period were examined using kernel analysis of filtered locations [24], using the locations between outward and return migration. The non-parametric fixed kernel density estimator was used to determine density contours. Kernel densities do not require serial independence of observations when estimating foraging ranges [25]. Kernel analyses were performed in a Lambert equal-area azimuthal projection centred on the South Pole using ArcGIS 9.3 (ESRI, Redlands, CA, USA) and the Hawth tool [26] (settings: scaling factor 10^6^, single parameter smoothing factor: 10^5^, raster cell size 5000). The distance travelled during outward and return migration trips was calculated in the same projection.

### Sex determination

The sex of each bird in this study was determined molecularly through PCR using primers 2550 and 2718 that amplify sections of the sex-linked chromo-helicase-DNA binding (CHD) gene according to [27]. DNA was extracted from 50 μl blood using a Qiagen DNAEasy blood purification kit (Qiagen, Hilden, Germany). Each reaction was carried out in 25 μl, containing 10 ng template DNA, 1 × PCR buffer, 0.1 mM DNTPs, 2.5 mM MgCl2, 0.2 μM of each primer and 0.1 U Taq polymerase (Firepol, Soilis Biodyne, Tartu). Thermocycling consisted of an initial denaturation step of 2 min at 94°C, followed by 35 cycles denaturation at 94°C for 30 s, annealing at 54°C for 30 s, extension at 72°C for 1 min, and ended with two expansion steps of 42°C for 1 min and 72°C for 10 min. PCR products were visualised on a 2% agarose gel, with a single band at ~650 bp indicating a male, and two bands at ~450 and ~650 bp indicating a female.

### Statistical data analyses and ecological niche modelling

Statistical analyses were conducted using SigmaStat 3.5 and R [28]. We tested for normality using Kolmogorov-Smirnov tests and by checking plots of the data. Means were calculated with their standard deviations. We found no significant difference in timing, location, distances or speed of travel between males and females in data visualisations or general linear models including site and sex as factors (all p > 0.05), therefore the data of both sexes were pooled.

The realized niches of the nonbreeding habitat of the two populations were modelled and extrapolated using the Bio-ORACLE data set [29], ETOPO2v2g bathymetry data (National Geophysical Data Center: http://www.ngdc.noaa.gov/mgg/global/relief/ETOPO2/) and MAXENT 3.3.3k (maximum entropy), as described previously [15]. We used eight non-redundant variables: ‘bathymetry’ (depth, m), ‘Mean chlorophyll’ (chlorophyll *a* mean, mg/m^3^), ‘Min chlorophyll’ (chlorophyll *a* minimum, mg/m^3^), ‘Min cloud cover’ (cloud cover minimum, %), salinity (salinity mean, PSU (practical salinity units)), ‘SST Fronts’ (sea surface temperature, range in °C over 3 × 3 grid cells), ‘Mean SST’ (sea surface temperature mean, °C) and ‘Min sea ice’ (presence/absence of sea ice during yearly minimum extent in February, categorical variable). The MAXENT program was run with the eight non-redundant variables for three data sets: (1) nonbreeding area of Kerguelen birds, (2) nonbreeding area of Falkland birds, (3) nonbreeding area of all thin-billed prions combined. MAXENT models were run with the following settings: logistic output format, resulting in values between 0 and 1 for each grid cell, where higher values indicate more similar climatic conditions and 50 replicate runs of random (bootstrap) sub-samples with 30% random test percentage. The results were summarized as the average of the 50 models, and the area under the receiver operating characteristic curve (AUC) was used for model evaluation. For projected suitable habitat maps, values below the 10th percentile training presence logistic threshold were removed.

### Stable isotope analyses

The isotopic method was already applied in the Southern Ocean, with δ^13^C values of seabirds indicating their latitudinal foraging habitats [18, 31] and their δ^15^N values increasing with trophic level [31]. Stable isotope values of feathers grown in the nonbreeding area (lower back/rump feathers in Kerguelen birds, a small segment of the inner vane of the innermost primary in Falkland birds) were collected during the retrieval of the geolocators. Becker et al. [32] observed a small but significant difference in δ^13^C values (0.6‰). The (dark) primary feathers were slightly depleted in δ^13^C, compared with the (white) breast feathers, and 0.6‰ is a typical difference when comparing black and white feathers [33]. However, the difference observed by Becker et al was much smaller than the difference observed here (1.8‰), and moreover, we used lightly pigmented feathers (grey lower back/ rump feathers and lightly pigmented parts of the primaries) in both cases, which should result in no or minimal differences caused by pigmentation. For Kerguelen birds, one body feather of each individual was cleaned of surface lipids and contaminants using a 2:1 chloroform:methanol solution for two min followed by two successive methanol rinses. Feathers were then air dried and homogenised by cutting them into small fragments. Tissue sub-samples were weighed (~0.4 mg) with a microbalance, packed in tin containers, and nitrogen and carbon isotope ratios were subsequently determined by a continuous flow mass spectrometer (Micromass Isoprime) coupled to an elemental analyser (Euro Vector EA 3024) at the LIENs laboratory from the University of La Rochelle, France. Replicate measurements of internal laboratory standards (acetanilide) indicated measurement errors < 0.15 ‰ for both δ^13^C and ^15^N values. Carbon and nitrogen isotope analyses of Falkland Island birds were carried out at the Scottish Universities Environmental Research Centre as described previously [34] on 0.65–0.7 mg feather aliquots, weighed in tin cups. Carbon and nitrogen isotope ratios were measured simultaneously by continuous-flow isotope ratio mass spectrometry (CF-IRMS) using a Costech Elemental Analyser (EA) linked to a Thermo Finnigan Delta Plus XP Mass Spectrometer. Two laboratory standards were analysed for every 10 unknown samples, allowing any instrument drift over a typical 14 hour run to be corrected. Based on internal standards (tryptophan), the analytical precision (± 1 SD) was estimated as ± 0.17 ‰ and ± 0.18 ‰ for δ^13^C and δ^15^N, respectively. All stable isotope ratios are expressed in δ notation as parts per thousand (‰) deviation from the international standards Vienna-Pee Dee Belemnite (carbon) and AIR (nitrogen).

## Results

### Outward migration

A total of 48 out of 50 thin-billed prions migrated away from their breeding area (i.e. the area used during the breeding season). The two exceptions were observed among Falkland birds in 2010 and 2011 that remained over the Patagonian Shelf during the non-breeding season. Outward migration took place immediately after either a failed breeding attempt or once the chick had fledged, resulting in two peaks of migration. For successful breeders, migration was 5 days earlier from Kerguelen Islands (mean 18 February, range 5 to 27 February) than the Falklands (mean 23 February, range 14 to 28 February, Table 2). Duration, distance and travel speed of outward migration were similar for the two populations (Table 2). Kerguelen birds moved westward along the Antarctic continent where they encounter easterly tail winds.

**Table 2:** Timing and duration of the migration and non-breeding areas in thin-billed prions from New Island, Falkland and Île Mayes, Kerguelen (means ± SD).

Parameter	Falkland	Kerguelen	test
**Outward migration**			
Departure date	54.0±4.1	48.8±6.1	t = 2.7, d.f. = 28**, *P* = 0.013**
Duration (days)	5.3±1.5	6.4±2.3	t = 1.5, d.f. = 28, *P* = 0.150
Distance (km)	3334±689	3371±645	t = 0.2, d.f. = 28, *P* = 0.878
Travel speed (km/day)	647±94	563±145	t = 1.8, d.f. = 28, *P* = 0.078
**Nonbreeding area**			
Duration (days)	81.5±18.5	208.4±49.2	t = 13.3, d.f. = 48**, *P* < 0.001**
95% kernel area (10^3^ km^2^)	1293±811	1220 ± 424	t = 0.3, d.f. = 52, *P* = 0.742
Centroid longitude	-7.2±8.6	10.7±9.2	t = 6.7, d.f. = 52**, *P* < 0.001**
Centroid latitude	-59.8±3.3	-53.2±3.5	t = 6.5, d.f. = 52**, *P* < 0.001**
Distance to colony (km)	3515±566	4214±662	t = 3.9, d.f. = 52**, *P* < 0.001**
**Return migration**			
Departure date	123.1±15.2	281.0±2.7	t = 37.0, d.f. = 41**, *P* < 0.001**
Duration (days)	10.0±3.4	4.8±1.9	t = 5.0, d.f. = 41**, *P* < 0.001**
Distance (km)	4586±1631	4187±1418	t = 0.7, d.f. = 41, *P* = 0.461
Travel speed (km/day)	469±116	909±211	t = 8.8, d.f. = 41**, *P* < 0.001**

Significant p-values are marked bold. Dates are given as Julian date (i.e. 1 Jan = 1). Two thin-billed prions from New Island without long-distance migration were excluded from the analyses. In the analyses of outward migration, we included all successful breeders (N = 13 for Falkland and 17 for Kerguelen). In the analyses of outward migration, we included only individuals with a clearly distinguishable, directed return track (N = 31 for Falkland and 13 for Kerguelen).

### Nonbreeding distribution

**Fig 1:**
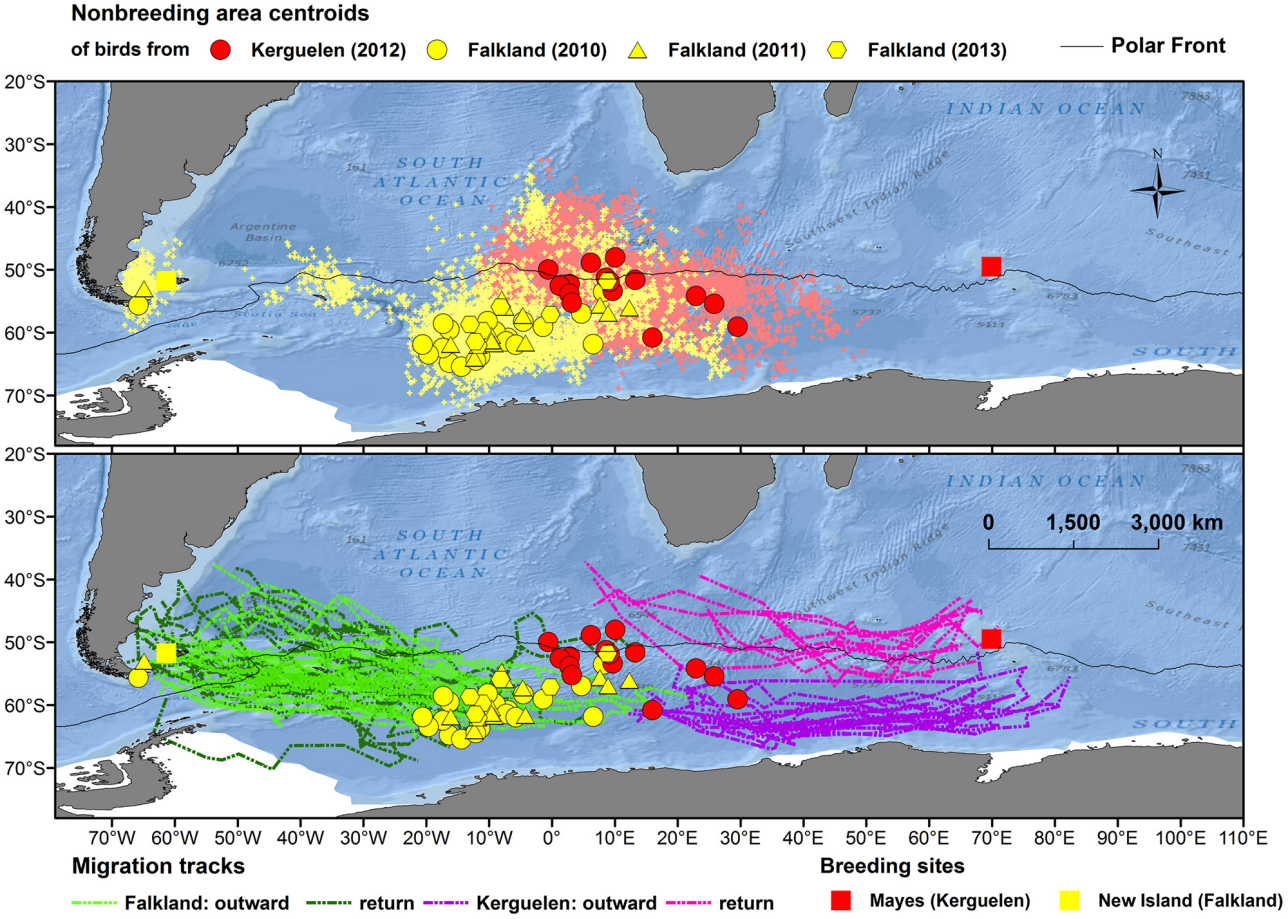
Migration of thin-billed prions from the Falkland and Kerguelen Islands, tracked using geolocators. In the upper map, small dots indicate positions recorded in the non-breeding area, while large symbols show the breeding colonies (squares without black margins) and the centroid positions of 95% kernels for each individual. In the lower map, outward and return journey tacks are shown.

Both populations migrated to a previously described nonbreeding area, located roughly between 30°W and 30°E, and 50°S and 65°S (Fig 1). The areas (95% kernel) used by individual birds in the nonbreeding area (*i*.*e*. between outward and return migration) were extensive and their size did not differ between birds from Kerguelen and the Falkland Islands (Table 2). The centroids were located further northeast for birds from Kerguelen (Table 2). The mean distance between the nonbreeding area centroids and the breeding colony was 700 km further away for birds from the Falklands (Table 2).

**Fig 2:**
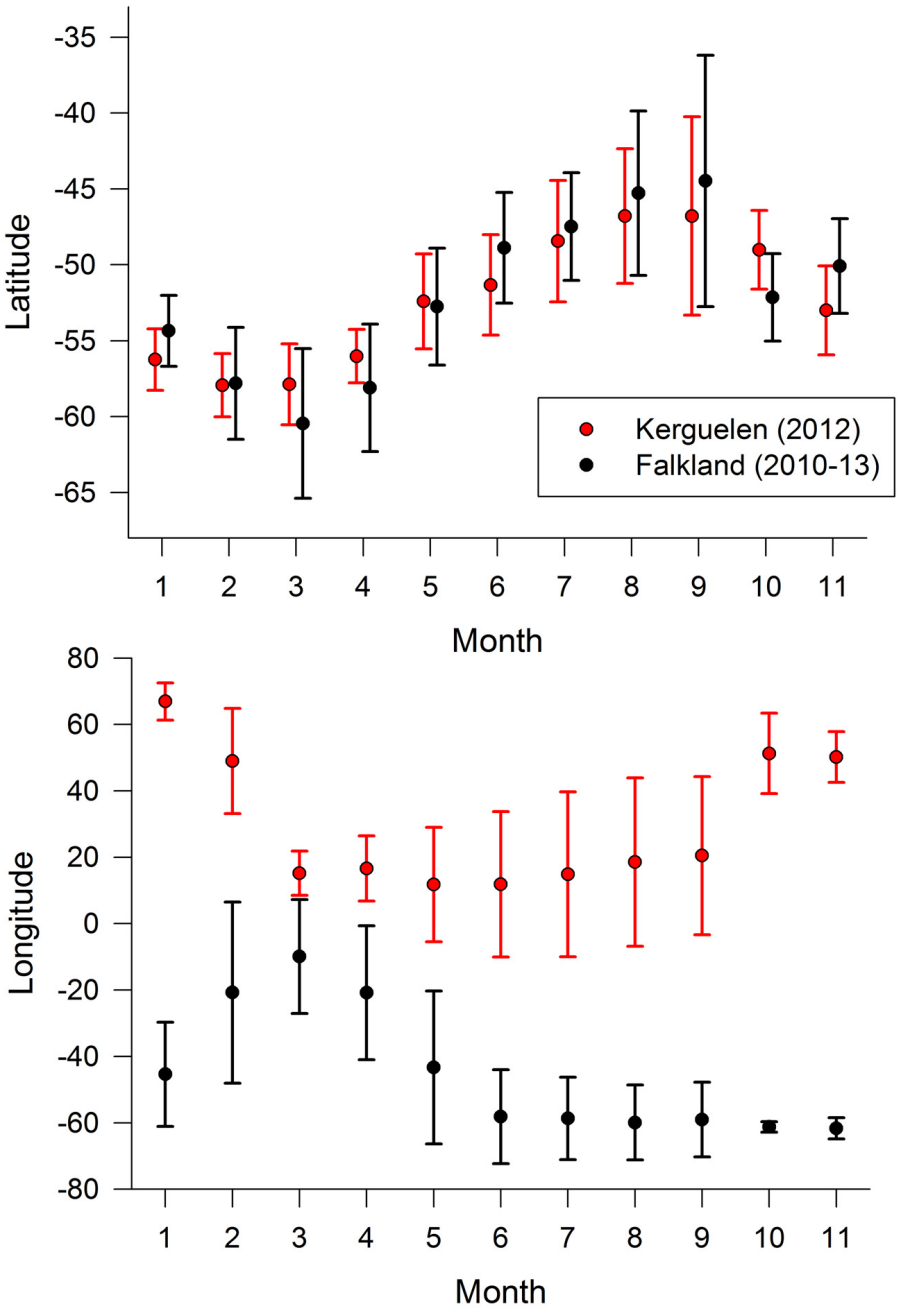
Year-round movements of thin-billed prions from the Falkland and Kerguelen Islands. Mean (± SD) latitudinal and longitudinal positions of thin-billed prions from the Falkland and Kerguelen Islands over the year.

Both populations showed a characteristic seasonal pattern of latitudinal distribution (Fig 2), with the lowest latitudes reached in March, i.e. during the early post-breeding period and the beginning of the presumed moult period. Although Kerguelen birds had similar patterns, they remained further north during March-April and showed a lesser degree of variability in their latitudinal range (Fig 2).

**Fig 3:**
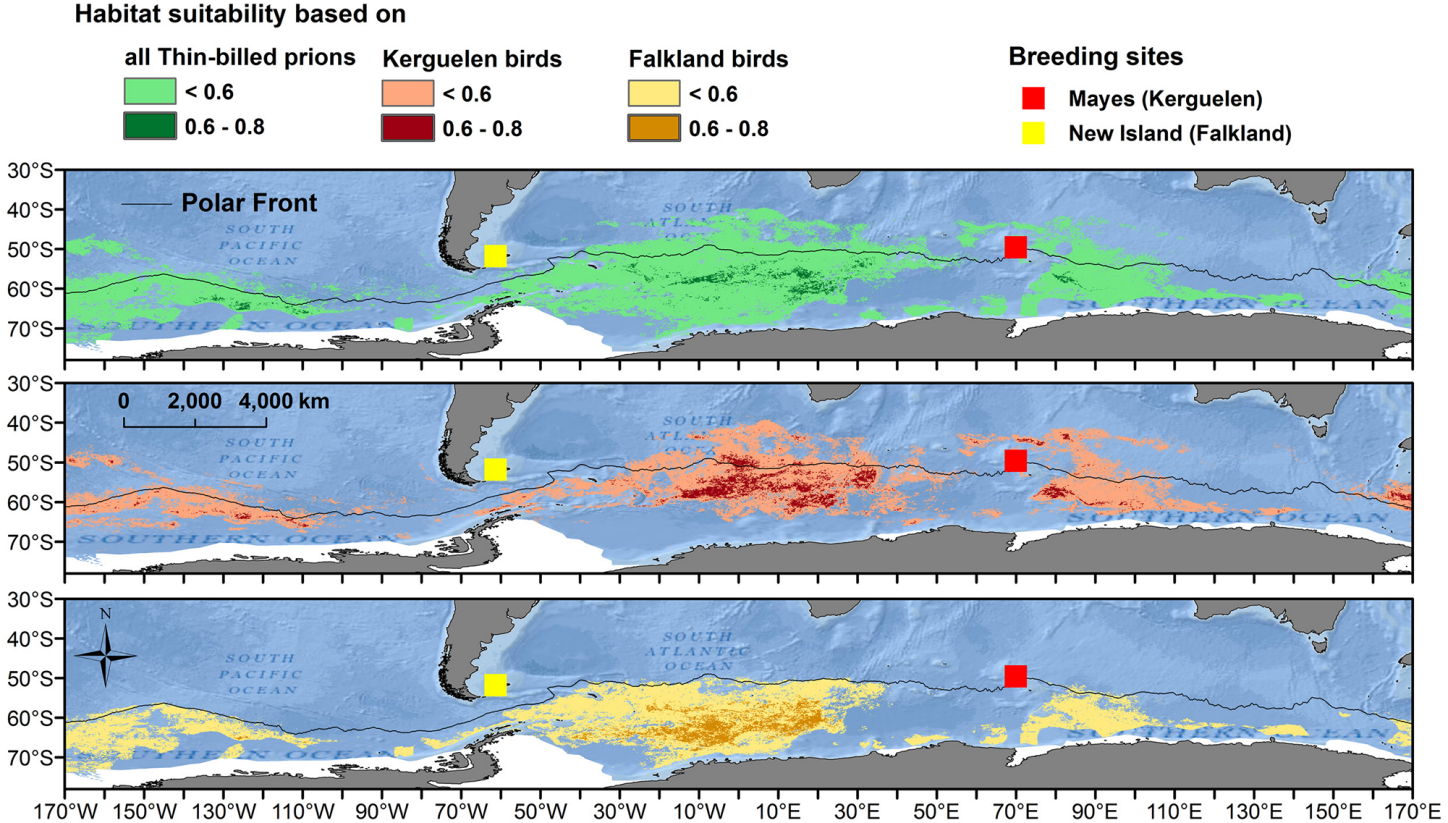
Habitat suitability models of thin-billed prions from the Falkland and Kerguelen Islands. Habitat values from MaxEnt models of the nonbreeding distribution of thin-billed prions from the Falkland and Kerguelen Islands. Values below the 10th percentile training presence logistic threshold (see Table 1) were omitted.

MAXENT models achieved AUC values that indicated good model fitting (Table 3). Sea surface temperature (SST) and mean chlorophyll concentration were the most important parameters, and were highly important in all three models after the permutation tests. Other key parameters were minimum cloud cover (only for the nonbreeding area of birds form Kerguelen) and salinity (S2 Fig). The most suitable habitat during the nonbreeding season was found exclusively south of the Polar Front (PF) for birds from the Falklands, while more northern areas were predicted for thin-billed prions from Kerguelen during the non-breeding period (Fig 3). When threshold values were applied (Table 3), the potential nonbreeding season distribution included areas in the Pacific and Indian Ocean.

**Table 3:** Estimates of model fit and relative contributions of the environmental variables to the MaxEnt model, normalized to percentages (values over 10% are marked bold), for the non-breeding period for the two populations of thin-billed prions so separately, and combined.

Model parameter	Falkland	Kerguelen	All
# cells	3163	3296	6459
Test AUC	0.845	0.837	0.765
10th percentile training presence logistic threshold	0.322	0.373	0.391
**Permutation importance**			
Bathymetry	3.5	2.3	3.0
Mean chlorophyll	**24.3**	**18.1**	**20.2**
Min chlorophyll	6.3	3.4	5.3
Min cloud cover	6.8	**42.4**	**22.0**
Salinity	**17.2**	**13.9**	**15.8**
SST fronts	0.2	1.1	0.6
Mean SST	**37.5**	**16.6**	**29.9**
Min sea ice	4.2	2.3	3.3

For the estimate of permutation importance, for each environmental variable in turn, the values of that variable on training presence and background data are randomly permuted. The model is re-evaluated on the permuted data, and the resulting drop in training AUC is shown in the table, normalized to percentages. Values shown are averages over 50 replicate runs. # cells: the number of cells with training samples.

### Return migration

Most Falkland birds remained at the nonbreeding area for 2–3 months, while birds from Kerguelen spent nearly 7 months there (Table 2). Consequently, Falkland birds returned to the area around their breeding grounds 5 months earlier (mean 5 May, range 14 April to 16 June) than Kerguelen birds (mean 7 October, range 29 September to 10 October, Table 2). The return of Kerguelen birds was very synchronous (starting within 12 days), short and fast, while the Falkland birds took twice as much time and travelled at half the speed (Table 2), probably because Kerguelen birds moved eastward on their return, making use of westerly tail winds, whereas Falkland birds were flying against the general westerlies flow.

### Annual variation

Among thin-billed prions from the Falklands, we found no annual differences in the outward migration or the position of the target area (Table 4). The birds also started the return migration on similar dates each year, and spent a similar number of days on the return migration. Slight inter-annual differences were observed in the return migration speed and the length of the track (Table 4), indicating that birds varied in their straightness of travel.

**Table 4:** Inter-annual comparison of the outward and return migration in thin-billed prions (means ± SD) from New Island, Falkland Islands.

Parameter	2010	2011	2013	test
**Outward migration**				
Departure date	54.2±3.3	53.9±4.9	-	t = 0.1, d.f. = 11, *P* = 0.898
Duration (days)	5.8±2.1	4.9±0.7	-	t = 1.1, d.f. = 11, *P* = 0.275
Distance (km)	3484±975	3204±340	-	t = 0.7, d.f. = 11, *P* = 0.490
Travel speed (km/day)	624±114	667±78	-	t = 0.8, d.f. = 11, *P* = 0.446
**Nonbreeding area**				
Duration (days)	79.8±17.5	80.1±22.4	90.6±12.9	ANOVA, F_2,32_ = 0.70, *P* = 0.505
95% kernel area (10^3^ km^2^)	1363±866	1399±869	907±437	ANOVA, F_2,36_ = 0.97, *P* = 0.387
Centroid longitude	-9.0±8.7	-4.9±8.9	-6.8±8.2	ANOVA, F_2,36_ = 0.87, *P* = 0.428
Centroid latitude	-60.8±3.0	-59.2±3.2	-58.1±3.5	ANOVA, F_2,36_ = 2.30, *P* = 0.115
Distance to colony (km)	3345±520	3675±574	3618±234	ANOVA, F_2,32_ = 1.72, *P* = 0.193
**Return migration**				
Departure date	123±14	126±18	115±11	ANOVA, F_2,28_ = 0.88, *P* = 0.427
Duration (days)	11.0±3.2	10.0±3.6	7.0±1.9	ANOVA, F_2,28_ = 2.9, *P* = 0.071
Distance (km)	5466±1305 ^a^	3943±1715 ^b^	3361±849 ^b^	**ANOVA, F**_**2,28**_ **= 5.90, *P* = 0.007**
Travel speed (km/day)	522±130 ^a^	390±68 ^b^	486±51 ^a,b^	**ANOVA, F**_**2,28**_ **= 5.31, *P* = 0.011**

Significant p-values are marked bold. Homogenous subsets in statistically significant comparisons are marked with the same superscript letter (a or b). Dates are given as Julian date (i.e. 1 Jan = 1). In the analyses of outward migration, we included only successful breeders (N_2010_ = 6, N_2011_ = 7). Nonbreeding area: N_2010_ = 19, N_2011_ = 13, N_2013_ = 7, Return migration: N_2010_ = 15, N_2011_ = 11, N_2013_ = 5.

### Feather stable isotopes

**Fig 4:**
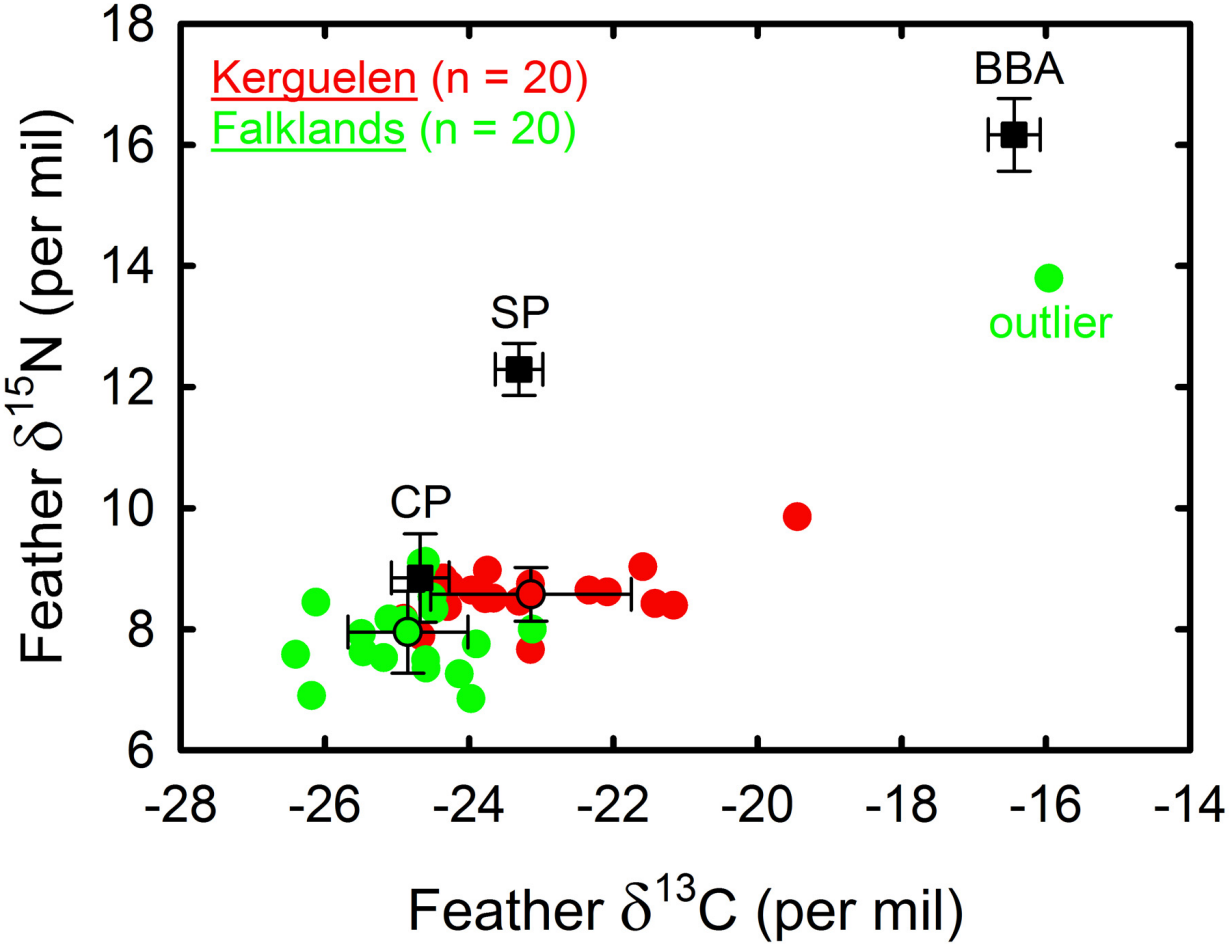
Stable isotope analyses of thin-billed prions from the Falkland and Kerguelen Islands. Feather δ^13^C and δ^15^N values of thin-billed prions from the Falklands (green) and Kerguelen Islands (red) grown in the nonbreeding area in 2010 and 2012, respectively (N = 20 per year). Note one thin-billed prion from the Falklands with very high stable isotope values (marked “outlier”). This bird did not migrate, but spent the whole nonbreeding season on the Patagonian Shelf. The signature of chick feathers of cape petrel (CP) and snow petrel (SP) from Adélie Land illustrate the δ^13^C values of species known to forage in high-Antarctic waters where they feed primarily on crustaceans and fish, respectively [53], authors’ unpublished data); the signature of chick down of black-browed albatross from New Island refers to a species that forage over the Patagonian shelf and feed on high trophic level prey [54, 55].

With regard to the feathers grown in the non-breeding season, one thin-billed prion from the Falklands that spent the winter on the Patagonian Shelf clearly separated from other studies birds, had high (relatively enriched) stable isotope values (δ^13^C = -16.0 ‰, δ^15^N = 13.8 ‰, Fig 4). Of the remaining birds, all of which had migrated, those from Kerguelen had higher δ^13^C values (-23.1 ± 1.4 ‰) than birds from the Falklands (-24.9 ± 0.8 ‰, t = 4.6, d.f. 37, *P* < 0.001, Fig 4). Feather δ^15^N values also differed between the two populations (Kerguelen: 8.6 ± 0.4 ‰, Falklands: 8.0 ± 0.7 ‰, t = 3.4, d.f. 37, *P* = 0.002, Fig 4). With an isotopic estimation of the Polar Front at -21.2 ‰ for feathers [30], all but one bird from the Falklands and all but one bird from Kerguelen Islands moulted at the Polar Front and further south, with most prions renewing their feathers in high-Antarctic waters.

## Discussion

We used geolocators to compare distributions of thin-billed prions from the two largest populations of the species, namely in the Falkland and Kerguelen Islands. We found that the two populations located more than 8,000 km apart used common moulting grounds located in Antarctic waters halfway between the two sites. These common grounds were reached by most individuals in autumn just after breeding. We observed similar migration patterns and phenologies across 3 years of data collection for Falkland birds. We further found a species-specific latitudinal pattern, albeit with a preference for slightly more southern latitudes during moult in thin-billed prions from the Falkland Islands.

### Timing of migration

The most striking differences between the two populations were the length of stay in the eastern Atlantic sector of the Southern Ocean, and consequently the timing of homeward migration. The outward migration of both populations was relatively straight and immediately after the breeding season or failed breeding attempt. However, whereas Kerguelen birds remained in the same broad sector to winter, Falkland birds moved back across the Atlantic to winter close to the breeding grounds over the Patagonian Shelf, indicating that the two populations have contrasting wintering strategies (Table 2, Fig 2 lower panel). Most thin-billed prions from the Falkland Islands only spent just over 80 days on average in the eastern Atlantic sector of the Southern Ocean. The δ^13^C values of first primaries indicated that birds moult there and, hence, the primarily goal of the fall movements of Falkland birds is to migrate to high-Antarctic waters to moult. In a previous study, data on progressively moulted feather series of primaries 1 to 10 indicated that most thin-billed prions from the Falkland Islands spent the whole feather moult time in one latitudinal area, only one of five birds had increasing δ^13^C values indicating movement to lower latitudes during moult [35].

In contrast to Falkland birds, thin-billed prions from Kerguelen remained in the eastern Atlantic sector of the Southern Ocean for over 200 days, on average. This extended period includes the moult period, taking place during the 2–4 months following the breeding season [36], and the rest of the non-breeding period in winter. Then birds return to the breeding site at the beginning of the courtship period, in October.

Previous studies of seabird migration have mainly concentrated on species-specific migration strategies. For example, thin-billed prions and closely related Antarctic prions (*Pachyptila desolata*, Gmelin 1789) from the south-west Atlantic have divergent patterns of migration, resulting in nearly complete spatial segregation [15]. In another example of related species, eastern and northern rockhopper penguins (*Eudyptes chrysocome filholi* Forster, 1781 and *E*. *moseleyi* Mathews & Iredale, 1921) overlapped in their spatial distribution in the Indian Ocean, but avoided significant overlap through a temporal delay of two months [37].

However, some studies have also compared the migration strategies between different populations of the same seabird species. North Atlantic and Mediterranean populations of Cory’s shearwaters *Calonectris diomedea* (Scopoli, 1769) used three common wintering areas, associated with up-welling systems of the tropical and southern Atlantic [5]. Together with the present study, this suggests that the use of wintering areas by several populations, found at large distances from the breeding colonies, may also occur in other pelagic seabirds. The present study is especially striking because the two populations arrive at the common breeding grounds from opposite directions. The routes taken by Kerguelen birds appear to have been selected so birds use favourable winds on both the outward and return journey. They first travelled westward along the Antarctic continent where they use the prevailing easterly winds, and then travelled further north on their return journey using the westerly wind flow. Other petrels are also known to select favourable wind conditions on their migration [38, 39]. In contrast, the routes of Falkland birds do not appear as optimal, showing that the location of common moulting grounds has led to contrasting migratory strategies for the separated colonies.

The concomitant use of GLS and SI allowed us to examine the spatio-temporal location of the occurrence of seabird moult, and suggests that the wintering areas of Falkland and Kerguelen thin-billed prions overlapped only in a small part of the moulting areas, and that the two populations had distinct distributions with different habitat characteristics. Likewise, neighbouring populations of diving seabird species often avoid overlap in winter areas (*e*.*g*. eastern rockhopper penguins [37], southern rockhopper penguins [40]). This suggests a need for resource partitioning operating at the population level in seabirds, even in winter, as has been shown for the breeding season when chick-provisioning seabirds are more constrained by central-place foraging [41].

**Fig 5:**
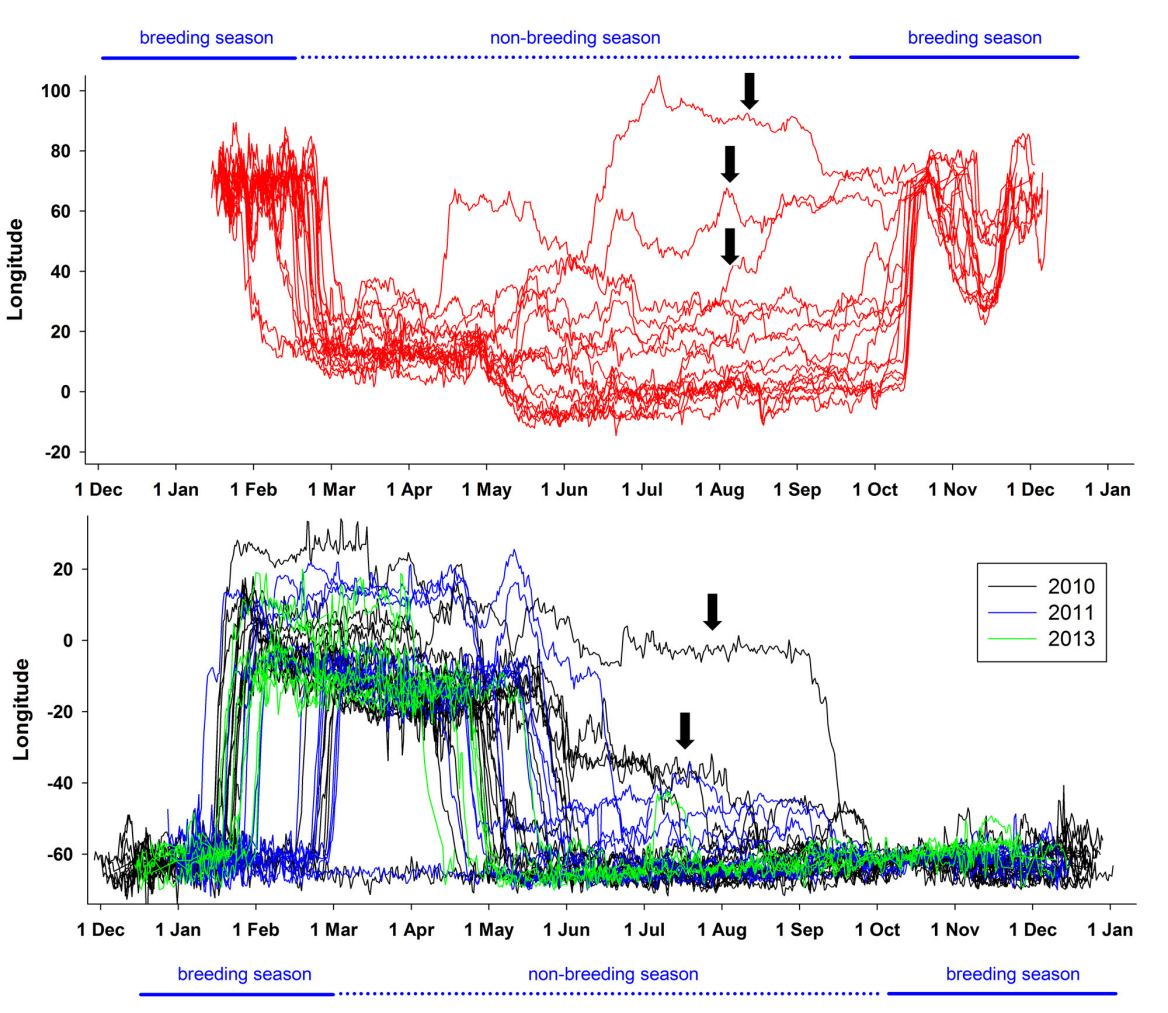
Individual variability of year-round longitudinal positions of Thin-billed prions. Birds were tracked using geolocators, from Kerguelen (upper panel, in red) and the Falkland Islands (lower panel). Birds exhibiting unusual migration timing are marked with arrows.

In addition to the population differences, we also observed individual differences in migration (Fig 5). Two Falkland individuals did not move to the eastern Atlantic to moult, but remained over the Patagonian Shelf. Thus, the Falkland population exhibits within-population migratory dimorphism similar to partial migration, with resident and migratory individuals. This has also been suggested using stable isotope analysis [18]. However, stable isotope data also indicated that while most individuals repeated their choice of winter area, individuals can also switch between the two strategies from year to year [35], thus demonstrating phenotypic plasticity rather than genetically fixed strategies.

Individual variability in winter phenology was further underscored by the large spread in return times in both populations (Fig 5). For example, one Falkland bird in 2010 remained in the eastern Atlantic sector of the Southern Ocean until early September (Fig 5, marked with an arrow). An exception to the general movement pattern was also seen in one of 15 Kerguelen birds. This bird first migrated to the common moulting area, but had already migrated back to waters south of Kerguelen from 4 to 10 June, and from there, carried on north-eastward, reaching a most easterly point at 105°E, 41°S on 5 July 2012. The bird remained far to the east throughout July and August, before moving into waters around Kerguelen in September (Fig 5, marked with an arrow). It has been suggested that various factors, such as competition for resources, predation risk and intraspecific niche diversity, may act synergistically to create complex patterns of migratory polymorphism within populations [42]. A degree of phenotypic plasticity in the phenology and site choice of avian migration is crucial for the ability of organisms to respond to naturally occurring environmental variability, and to climate change. Thus, selection may favour highly plastic individual migratory phenologies in variable or changing environmental conditions [43]. This should be even more evident in long-lived birds such as thin-billed prions.

### Moulting area

Most Falkland spend the first part of the winter in the eastern part of the Atlantic and we can conclude from the stable isotope data that primary moult occurs in this area (e.g. see also [35]). The moulting areas found in the present study are also used by other species of Procellariiformes for moult, such as Light mantled sooty albatrosses *Phoebetria palpebrata* Forster, 1785 (HW unpublished), suggesting that this sector of the south east Atlantic could be a major site for several Southern Ocean species. This is an open water area of 2000–4000 m depth. Why it is used during moult by populations arriving from western Atlantic and Indian Ocean is not well known, but it is likely that prey concentrations play a major role, especially during this energetically demanding period. Thin-billed prions feed predominantly on zooplankton during the breeding season [16, 17, 22], and feather stable isotope values indicated that low trophic levels are also maintained during moult (Fig 4).

Thin-billed prions feed predominantly on zooplankton in the size range of 8–15 mm (mainly euphausiids and amphipods). Although the Southern Ocean is dominated by zooplankton of two size classes, *i*.*e*. <10 mm (*e*.*g*. copepods) and 20–50 mm (*e*.*g*. Antarctic krill *Euphausia superba*, Dana, 1850, [44]), the 4–28 mm large hyperiid amphipod *Themisto gaudichaudii* (Guérin-Méneville, 1825) is an abundant zooplankter in the intermediate size class, and was found to be the most important prey item for thin-billed prions from both the Falkland [22] and Kerguelen Islands [16, 17]. *Themisto gaudichaudii* is a cold-water species distributed in the southern hemisphere and has been described as a voracious carnivore often occurring in enormous swarms. In the Southern Ocean it is found within the West Wind Drift from 39°S—63°S, including the southern Patagonian and Kerguelen waters [45, 46]. In the Discovery Investigations (1925–1951), *Themisto gaudichaudii* were particularly abundant in December and in March, when they were concentrated in two high-density belts located south and north of the Antarctic Polar Front, centred at 46–47°S and 53°S [47]. This matches the winter distribution of the thin-billed prions, especially those from Kerguelen (*e*.*g*. Fig 2). However, over the past decades, Southern Hemisphere westerlies have shifted poleward and increased in intensity [48], leading a southward shift of the Polar Front [49]. In fact, while Kane [47] registered the Polar Front south of South Africa at 49–50°S, it was given at 52°S in [50]. Therefore, distribution of zooplankton may also be shifted, and thin-billed prions from the Falklands with their more southerly distribution may also be feeding mainly on *Themisto gaudichaudi*. Alternatively, krill and copepods may be taken further south [44]. During breeding, thin-billed prions from the Falklands may use copepods as replacement when amphipods and krill are scarce [22], and birds from Kerguelen prey upon Antarctic krill when performing long trips [16]. Comparisons of recent and historic feathers suggested that both populations of thin-billed prions exhibited a latitudinal change in the moulting grounds towards more polar waters over the last decades [17, 18]. This can be explained with the results of the present study, as both populations moult within the same area. The fact that both populations show similar latitudinal movements over the winter (Fig 2) also suggests that both populations respond to similar cues or movements of their prey.

Finally, the question why Kerguelen birds remain in the same broad sector to winter, whereas Falkland birds do not, might be explained with differences in prey abundance. Better foraging conditions around the Falklands islands than around Kerguelen were already suggested for the breeding season, based on higher chick-provisioning rates [16, 51]. If these differences persist in winter, then we would expect Kerguelen birds to remain in their winter quarters as long as possible. In contrast, Falkland birds may benefit from spending the winter over the Patagonian Shelf, which offers a range of abundant prey in waters over the extensive shelf area and shelf slope, and the extensive and highly productive confluence zone of the Brazil Current with the Falkland/Malvinas current. In addition to these boundary currents, the extent of the Patagonian Shelf allows for the development of mesoscale fronts [52], including a number of year-round and seasonal tidal fronts, such as the Bahía Grande Front and the Valdés Front which support, among other zooplankton, populations of the favoured prey, *Themisto gaudichaudii*. Furthermore, the presence in winter may enable thin-billed prions from the Falklands to better adjust the timing of reproduction to the prevailing conditions in this highly seasonal ecosystem.

## Conclusions

The results presented here show remarkable similarities and differences in migration strategies of two spatially separated populations of a small pelagic seabird. Arriving from opposite directions, they reach a common moulting ground in autumn, where they show some spatial segregation during the moulting season (MarchMay). During winter (JuneSeptember), Falkland birds leave this area and thus spatial segregation is complete except for very few individuals that differ in their phenology from the rest of the population (*e*.*g*. the outlier on Fig 4). Together with other studies, the behaviour of these individuals suggests selection for high phenotypic plasticity, in order to cope with the variability of the oceanographic conditions and thus, distribution of prey. While opposing migratory directions may act to promote population differentiation and possibly speciation, this process may be offset by phenotypically plastic individuals whose irregular distribution could result in successful exchange and gene flow between the populations.

## Supporting Information

S1 Fig Example of longitudinal positions of one Thin-billed prion from Kerguelen. **Upper panel**: Year-round positions, used to show the main phases of the yearly cycle. All longitude values (blue dots; lon_all) were overlayed with filtered longitude values (red dots; lon_filtered), where any unrealistic positions—either associated with interference to light curves at dawn or dusk, or in temporal proximity to equinoxes—were excluded. **Lower panel**: Focused on return migration of the same individual. The timing of migration was determined from directed longitudinal movements that finished at or beyond the breeding colony longitude. These were clearly distinguished in all individuals.
(DOCX)

S2 Fig Response curves for the four key parameters in the MaxEnt models of nonbreeding areas of thin-billed prions from the Falkland and Kerguelen Islands. (DOCX)
