# Supplementary material for: Half a World Apart? Overlap in Nonbreeding Distributions of Atlantic and Indian Ocean Thin-Billed Prions
Source: PLoS One. 2015 May 27;10(5):e0125007. doi: 10.1371/journal.pone.0125007 (PMC4446212; doi:10.1371/journal.pone.0125007)

**Figure S1.** Example of longitudinal positions of one Thin-billed prion from Kerguelen. **Upper panel**: Year-round positions used to show the main phases of the yearly cycle. All longitude values (blue dots; lon_all) were overlayed with filtered longitude values (red dots; lon_filtered), where any unrealistic positions - either associated with interference to light curves at dawn or dusk, or in temporal proximity to equinoxes - were excluded.

**Lower panel**: Focused on return migration of the same individual. The timing of migration was determined from directed longitudinal movements that finished at or beyond the breeding colony longitude.


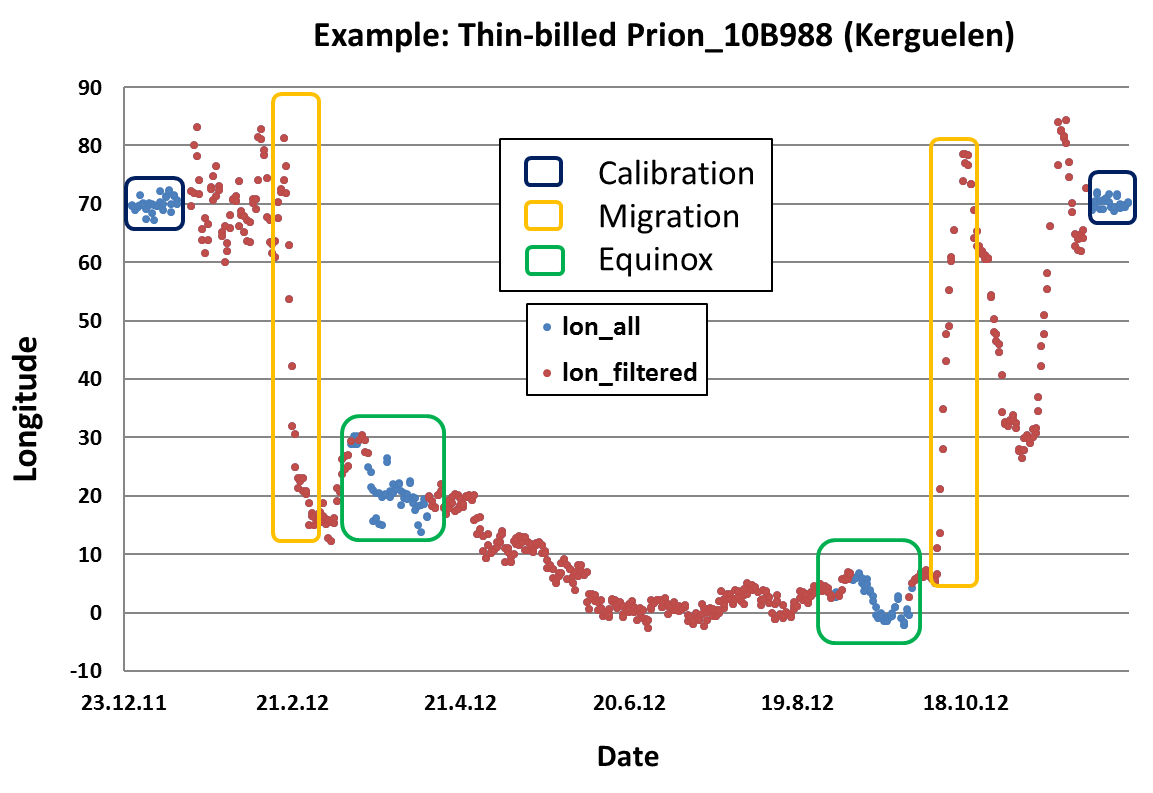

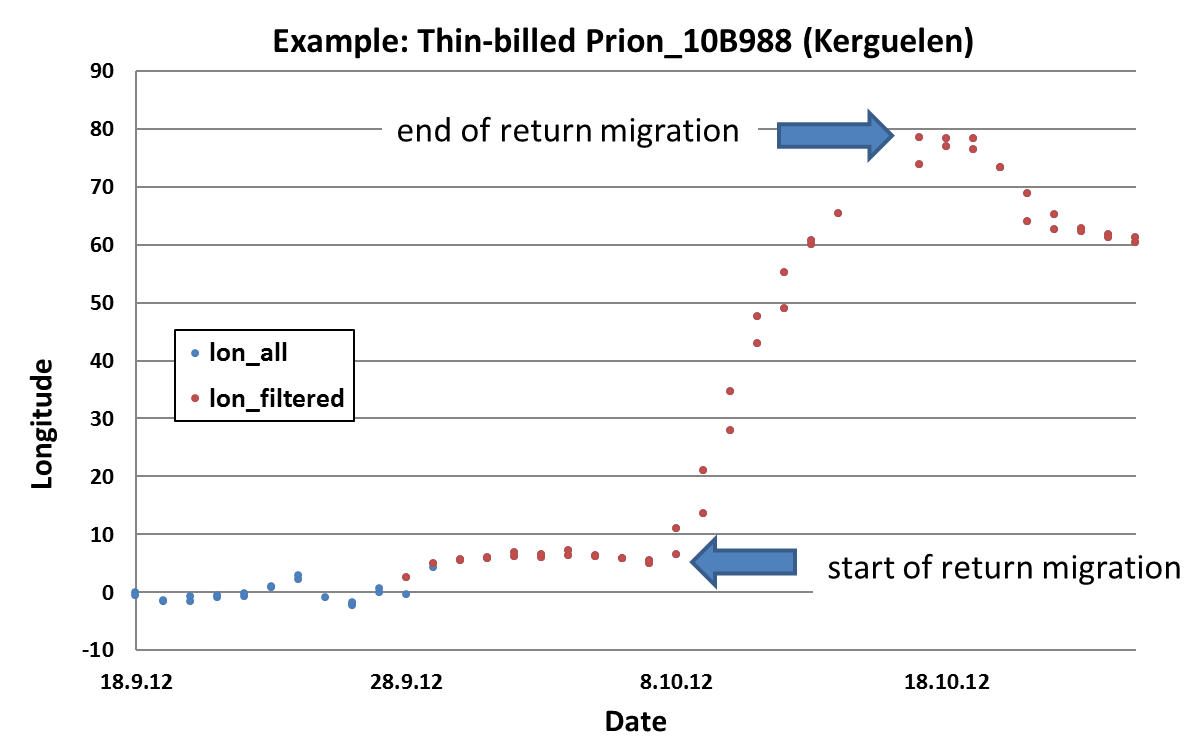

Supplement: S1 Fig — Upper panel: Year-round positions, used to show the main phases of the yearly cycle. All longitude values (blue dots; lon_all) were overlayed with filtered longitude values (red dots; lon_filtered), where any unrealistic positions—either associated with interference to light curves at dawn or dusk, or in temporal proximity to equinoxes—were excluded. Lower panel: Focused on return migration of the same individual. The timing of migration was determined from directed longitudinal movements that finished at or beyond the breeding colony longitude. These were clearly distinguished in all individuals. (DOCX) [file pone.0125007.s001.docx]
