# Supplementary material for: Half a World Apart? Overlap in Nonbreeding Distributions of Atlantic and Indian Ocean Thin-Billed Prions
Source: PLoS One. 2015 May 27;10(5):e0125007. doi: 10.1371/journal.pone.0125007 (PMC4446212; doi:10.1371/journal.pone.0125007)

**Figure S2.** Response curves for the four key parameters in the MaxEnt models of nonbreeding areas of thin-billed prions from the Falkland and Kerguelen Islands.


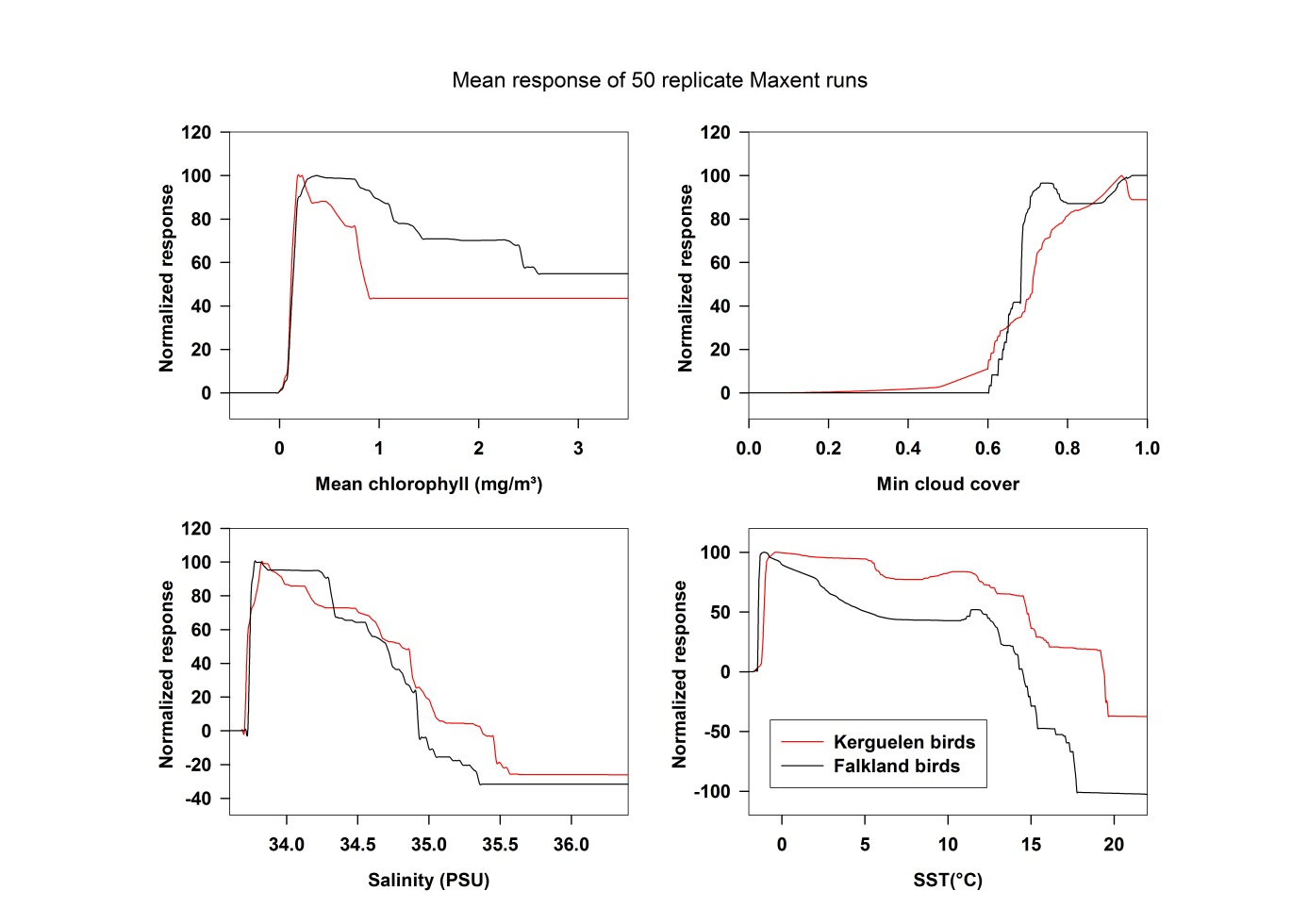

Supplement: S2 Fig — (DOCX) [file pone.0125007.s002.docx]
